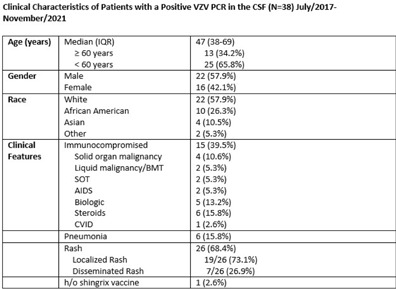# Zoster on the brain: Clinical characteristics of patients PCR positive for varicella-zoster virus in cerebrospinal fluid and implications for transmission base

**DOI:** 10.1017/ash.2022.171

**Published:** 2022-05-16

**Authors:** Mahmoud Al-Saadi, Michael Haden, Nora Colburn, Shandra Day, Christina Liscynesky

## Abstract

**Background:** Transmission-based precautions against varicella-zoster virus (VZV) in healthcare settings are determined by the extent of rash (localized vs disseminated) and the immune status of the host. At our facility, immunocompetent patients with localized disease are placed in standard precautions whereas patients with disseminated disease and/or immunocompromised status are placed in airborne and contact isolation. The use of molecular diagnostics has increased recently, and patients can have a PCR positive for VZV in cerebral spinal fluid (CSF) without evidence of pneumonia or disseminated rash. These patients are classified as disseminated disease, but it is unlikely that they are spreading VZV via respiratory aerosols in the absence of other symptoms. Infection prevention guidance is limited in this situation, and these patients may be in unneeded isolation, with the potential for adverse patient effects and overutilizing PPE resources. We have described the clinical characteristics of patients with a PCR positive for VZV in CSF, and we evaluated the risk for transmitting VZV via airborne aerosols. **Methods:** A retrospective, single-center chart review was performed on all patients admitted with a PCR positive for VZV in CSF between July 2017 and November 2021. Chart review was performed to gather data regarding clinical presentation, patient characteristics, and risk factors. **Results:** In total, 38 patients were identified who had a PCR positive for VZV in CSF; 22 (57.9%) were male and 16 (42.1%) were female. The median age was 47 years (IQR, 38–69). Also, 15 patients (39.5%) were immunocompromised. Moreover, 26 patients (68.4%) had a rash; 19 (50%) had localized rash; and 7 (18.4%) had disseminated rash involving ≥3 dermatomes. However, 12 patients (31.5%) had neither rash nor pneumonia. Furthermore, 5 patients (13.1%) had PCR positive for VZV in CSF and developed rash within the following 2–7 days (2 with disseminated rash). In addition, 6 patients (15.8%) had pneumonia. Of the 6 patients with pneumonia, 4 (10.5%) were immunocompromised and 3 (7.9%) were above 65-year-old. 32 patients (84.2%) were kept in airborne and contact precautions. 1 (2.6%) patient had a documented record of at least 1 dose of Shingrix vaccine. **Conclusions:** Most patients with a PCR test positive for VZV in the CSF were not immunocompromised and did not have evidence of disseminated rash or pneumonia. The risk of airborne transmission of VZV via small aerosols appears to be low in patients with a PCR test positive for VZV in the CSF without evidence of disseminated rash or pneumonia. Airborne isolation may not be required for many of these patients.

**Funding:** None

**Disclosures:** None

**Table:**